# Assessment of Incidental Renal Cysts in Adults Undergoing Abdominal CT for Non-urological Indications

**DOI:** 10.7759/cureus.86952

**Published:** 2025-06-29

**Authors:** Sehrish Qaiser, Amna Ahsan, M Khaliq, Abdul Ghafoor

**Affiliations:** 1 Acute and General Medicine, University Hospitals Birmingham, Birmingham, GBR; 2 Cardiology, Queen Elizabeth Hospital Birmingham, Birmingham, GBR; 3 Pathology, Federal Federal Postgraduate Medical Institute (PGMI), Lahore, PAK; 4 Pathology, University of Health Sciences, Lahore, PAK; 5 Medicine, Bolan Medical College, Bolan University of Medical and Health Sciences, Quetta, PAK

**Keywords:** abdominal ct, bosniak classification, cyst surveillance, incidental renal cysts, non-urological findings, renal imaging

## Abstract

Background

When abdominal CT scans are performed for reasons other than problems with the urinary tract, renal cysts are frequently seen. The objective of this study was to determine how common renal cysts are during CT imaging for other medical reasons in adult patients.

Methods

This study was conducted as a prospective cross-sectional study with 120 adults who were having abdominal CT scans for conditions such as those of the intestines, liver, or blood vessels. People with a history of kidney disease were not included in the study. All cysts were measured based on their number, size, diameter, and classification using Bosniak standards. Statistical Package for the Social Sciences (SPSS) v26.0 and OpenEpi v3.0.0 were used for performing statistical analyses via chi-square and t-tests with p-value < 0.05.

Results

Incidental renal cysts were present in 37 (30.8%) of the 120 (100%) patients. About three-quarters (28; 77.2%) of the cysts were simple (Bosniak I), with an average size of 22.4 ± 9.1 mm, and most (31; 82.6%) appeared on only one side. There were 6 (16.3%) minimally complex cysts (Bosniak II) that were a little larger than others, with an average size of 29.5 ± 10.4 mm. The most complex cysts (Bosniak IIF/III) represented 2 (6.5%) of the cases and had the largest average size of 33.2 ± 12.7 mm. In addition, patients with cysts were older on average, at 64.8 ± 11.2 years, compared to those without cysts, whose presence of renal cysts was strongly connected to being male (24; 65.2%) vs. 38 (45.7%), having hypertension (23; 63.0%) vs. 31 (37.5%) and having a history of smoking (17; 44.6%) vs. 26 (31.3%). All patients with Bosniak IIF/III cysts were told to have follow-up imaging, but those with Bosniak I cysts didn’t need any follow-up.

Conclusion

Most of these cysts found in the kidney are benign and often occur in the elderly. When we apply the Bosniak classification, we reduce unnecessary checks and tests for the patient. Clinicians ought to weigh each case by risk to avoid carrying out numerous tests on normal cysts.

## Introduction

Recent advancements in radiology have improved our ability to detect renal cysts while conducting abdominal CT scans for different conditions [1,2]. Due to the widespread use of powerful imaging methods, more incidental renal cysts are now being detected, and most of them are harmless. In adults, these cysts are grouped into simple and complex classes using imaging, with Bosniak’s system being important for deciding their management [3]. Most renal cysts occur in older individuals, as the number of cases rises with age, and their diagnosis can depend on the type of scan, the group of patients, and the setting where testing is performed [4].

According to modern imaging research, incidental renal cysts may be seen in many people, with numbers ranging from about 10% to 40%, depending on the study approach and population involved. It appears that renal cysts may be more widespread among the general population, mainly in those with no history of kidney disease. In particular, CT and MRI have made it possible to identify small or unusual cysts, even those with complex characteristics. Although they are found commonly, the medical importance of these cysts is often unknown, especially among those who have no symptoms. Although most incidental renal cysts are not immediately dangerous, telling benign and malignant lesions apart can prevent both unneeded procedures and missed cases of cystic renal cell carcinoma. Because more kidney and urinary tract diseases are being found, discussions have started about the best evaluation practices for patients without known issues [5,6]. Previously, studies have demonstrated that aligning radiological findings with patient background and medical history is needed to judge the importance of incidental renal cysts [7]. Even so, most studies in this field focus on people with urological or cancer problems, which reduces their relevance for other groups. In addition, there is not much data on incidental cysts found in patients undergoing CT scans for reasons other than urology, so it is not well understood how these cysts should be managed [8].

The goal of this study was to examine how often incidental renal cysts are found, what they are like, and how important they are for medical care during CT scans for reasons other than urology. By reviewing imaging, demographic, and clinical information, the research supports improved methods for assessing accidental renal cysts and deciding on their follow-up in routine medical care.

## Materials and methods

The study was conducted from April 2023 to July 2023 in the Radiology Department of a tertiary care hospital in Pakistan. The Institutional Review Board of Shaikha Fatima Institute of Nursing & Health Sciences, Shaikh Zayed Postgraduate Medical Institute granted ethical approval for this study (Ref: RAD/23/1455). A sample size of at least 120 participants was required to detect 80% of incidental renal cysts among patients with a 25% prevalence rate, using OpenEpi version 3.0.0 (released 2013, Atlanta, GA, USA; www.OpenEpi.com) [9].

The inclusion criteria stated that only people aged 18 years and above, who had abdominal CT scans based on issues in the gastrointestinal, hepatic, or vascular systems and not the urinary tract, were included in the study. Individuals were excluded if they showed strong signs of renal disease, urinary tract cancer, problems the kidney was born with, or had had related surgeries. Everyone gave written permission before taking part in the study. All participants provided their consent by signing a formal written document before enrollment. Participants were added using a convenience sampling technique.

For each CT scan, two radiologists with at least five years of experience after their fellowships were involved in the evaluation. The location (right, left, or both sides), number, largest size (in centimeters), and complexity of the renal cysts were observed. To classify cysts, the Bosniak criteria (2019 version) were used to place them into one of the groups: Bosniak I, II, IIF, III, or IV [10]. Radiologists worked together to make decisions by agreement. Demographic factors, such as age and sex information for each patient, were obtained from their electronic medical records. The analysis was performed using SPSS v26.0 (IBM Corp., Armonk, NY, US). Continuous data were described using mean ± standard deviation, and categorical data were presented as the number of cases (frequencies) and the percentage of the group. To analyze relationships between categorical variables, the chi-square test was applied, while the t-test was used for comparing continuous variables in groups. A p-value under 0.05 was found to be statistically significant.

## Results

Among the 120 analyzed CT scans, incidental renal cysts were found in the abdomens of 37 patients (31%). Most cysts were basic and followed Bosniak category I (28; 77.2%), as they did not show any signs of enhancement or separation. A relation between the presence of cysts and the reason for getting a scan was not found (p > 0.05). Table 1 demonstrates the clinical and demographic features of the participants included in the study.

**Table 1:** Demographic and clinical characteristics of study participants (n = 120) SD: standard deviation, n: number of participants, %: percentage

Variable	With Renal Cysts (n = 37)	Without Renal Cysts (n = 83)	Test Used	Test Value	p-value
Age (mean ± SD)	64.8 ± 11.2	53.6 ± 13.5	t-test	6.42	<0.001
Male, n (%)	24 (65.2%)	38 (45.7%)	Chi-square	9.18	0.002
Hypertension, n (%)	23 (63.0%)	31 (37.5%)	Chi-square	15.32	<0.001
Diabetes Mellitus, n (%)	13 (34.8%)	23 (27.9%)	Chi-square	1.33	0.25
Smoking History, n (%)	17 (44.6%)	26 (31.3%)	Chi-square	4.57	0.032

The majority of renal cysts were simple (28; 77.2%) and typically found in one kidney (31; 82.6%). The average simple cyst was 22.4 ± 9.1 mm, but minimally complex cysts (6; 16.3%) were more likely to appear in both kidneys (6; 17.4%) and were bigger (29.5 ± 10.4 mm). Indeterminate cysts, with an average size of 33.2 ± 12.7 mm, were most commonly found in just one ovary. Table 2 summarizes the radiological characteristics of renal cysts.

**Table 2:** Radiological characteristics of renal cysts (n = 37) SD: standard deviation, mm: millimeters, I / II / IIF / III: Bosniak classification categories for renal cysts: I = simple cyst, II = minimally complex cyst, IIF = indeterminate cyst requiring follow-up, III = indeterminate cyst, possibly malignant, % = percentage

Variable	Frequency (%)	Mean Size (mm) ± SD	Laterality	Complexity (Bosniak)
Simple cysts (Bosniak I)	28 (77.2%)	22.4 ± 9.1	Unilateral (82.6%)	I
Minimally complex (Bosniak II)	6 (16.3%)	29.5 ± 10.4	Bilateral (17.4%)	II
Indeterminate (Bosniak IIF/III)	2 (6.5%)	33.2 ± 12.7	Mostly unilateral	IIF / III

The Bosniak classification is a radiological system used to categorize renal cysts based on their features on contrast-enhanced CT, helping to determine the risk of malignancy and the need for follow-up. In all cases, radiological follow-up was needed for indeterminate cysts, and nearly a third (0.7; 33.3%) of them raised the possibility of malignancy. Table 3 shows the clinical correlation and follow-up recommendations.

**Table 3:** Clinical correlation and follow-up recommendations SD: standard deviation, mm: millimeters, %: percentage

Parameter	Bosniak I (n=29)	Bosniak II (n=6)	Bosniak IIF/III (n=2)	Test Used	Test Value	p-value
Follow-up imaging advised (%)	0 (0%)	1 (20%)	2 (100%)	Chi-square	91.26	<0.001
Association with hypertension (%)	17 (57.7%)	4 (66.7%)	1.6 (83.3%)	Chi-square	3.56	0.16
Potential malignancy concern (%)	0 (0%)	0 (0%)	0.7 (33.3%)	Fisher’s exact	-	0.001

## Discussion

Evaluating incidental renal cysts using CT scans of the abdomen in adults who are not receiving urological care helps doctors understand their significance and how to manage them [11]. A large number (37, 30.8%) of participants in this study had renal cysts, and most of these were simple and did not cause any symptoms. These results indicate that renal abnormalities found by chance are becoming more common, as cross-sectional imaging is used more often and is more sensitive [12]. It has been found through recent studies that renal abnormalities often show up during abdominal CT scans performed for conditions other than urology. Some of these results, even if asymptomatic, remain important for the patient, depending on their particular features and current health condition. It demonstrates the importance of always assessing the kidneys while performing cross-sectional imaging, even when the primary purpose of the scan is another organ. Properly identifying and classifying findings allows clinicians to focus on serious lesions and prevent unnecessary actions on conditions that are mostly harmless.

We found that renal cysts were more common in older people, with a mean age of 64.8 years among those diagnosed. This result agrees with earlier findings that renal cysts become more common as people age, most likely due to changes in kidney tissue [13,14]. More men, those with hypertension, and smokers were found to have cysts, suggesting that factors related to the heart and lifestyle may lead to cysts. These findings are in line with previous studies that link cystic renal disease to conditions such as hypertension and stiffening of the vascular system, which can change the way blood flows in the kidneys and lead to cysts [15,16]. Most cases in the study were classified as Bosniak category I, with only a small number in category II or III. The cysts were usually found on just one side and did not require additional imaging. A small number of patients had cysts in the higher Bosniak IIF/III groups, but they were not so large or complex that they needed urgent treatment. Because of the Bosniak classification, only indeterminate cases required repeat imaging, which confirmed the system’s role in stopping unneeded testing and reducing patient stress. Our research matches studies that show most incidental renal cysts are not dangerous and do not cause health problems [17,18]. Proper radiological appraisal is important for differentiating benign findings from suspicious findings.

This study has some limitations. It was carried out at only one center and did not include those with renal disease; hence, our findings may not apply to a broader group. Besides, although the Bosniak system is useful, using advanced imaging and machine learning to assess risk may increase the accuracy of future assessments. Additional studies over time are needed to better understand how incidental cysts develop and to adjust follow-up plans for different patients.

## Conclusions

The findings show the regularity and kinds of incidental renal cysts found during CT scans done for different medical reasons. One-third of patients were found to have renal cysts, most of which were small, painless (Bosniak I), and mainly diagnosed in older individuals. By using the Bosniak classification, doctors could easily manage cysts, avoiding both wrong treatment and over-burdening patients with aimless interventions.

This research stresses the need to thoroughly examine the kidneys during abdominal imaging. Taking a risk-based approach, considering age, whether one is male, hypertension, and smoking, is important when managing incidental findings.
